# SARS-CoV-2 treatment effects induced by ACE2-expressing microparticles are explained by the oxidized cholesterol-increased endosomal pH of alveolar macrophages

**DOI:** 10.1038/s41423-021-00813-6

**Published:** 2022-01-04

**Authors:** Zhenfeng Wang, Jiadi Lv, Pin Yu, Yajin Qu, Yabo Zhou, Li Zhou, Qiangqiang Zhu, Shunshun Li, Jiangping Song, Wei Deng, Ran Gao, Yuying Liu, Jiangning Liu, Wei-Min Tong, Chuan Qin, Bo Huang

**Affiliations:** 1 Department of Immunology & National Key Laboratory of Medical Molecular Biology, Institute of Basic Medical Sciences, Chinese Academy of Medical Sciences (CAMS) & Peking Union Medical College, Beijing, 100005 China; 2 NHC Key Laboratory of Human Disease Comparative Medicine, Beijing Key Laboratory for Animal Models of Emerging and Remerging Infectious Diseases, Institute of Laboratory Animal Science, CAMS and Comparative Medicine Center, Peking Union Medical College, Beijing, China; 3 State Key Laboratory of Cardiovascular Disease, Fuwai Hospital, National Center for Cardiovascular Diseases, CAMS and Peking Union Medical College, Beijing, China; 4 Department of Pathology, Institute of Basic Medical Sciences, CAMS and Peking Union Medical College, Beijing, China; 5 Department of Biochemistry & Molecular Biology, Tongji Medical College, Huazhong University of Science & Technology, Wuhan, 430030 China

**Keywords:** SARS-CoV-2, microparticles, Alveolar macrophages, Endosomes, Lysosomes, Alveolar macrophages, Viral infection

## Abstract

Exploring the cross-talk between the immune system and advanced biomaterials to treat SARS-CoV-2 infection is a promising strategy. Here, we show that ACE2-overexpressing A549 cell-derived microparticles (AO-MPs) are a potential therapeutic agent against SARS-CoV-2 infection. Intranasally administered AO-MPs dexterously navigate the anatomical and biological features of the lungs to enter the alveoli and are taken up by alveolar macrophages (AMs). Then, AO-MPs increase the endosomal pH but decrease the lysosomal pH in AMs, thus escorting bound SARS-CoV-2 from phago-endosomes to lysosomes for degradation. This pH regulation is attributable to oxidized cholesterol, which is enriched in AO-MPs and translocated to endosomal membranes, thus interfering with proton pumps and impairing endosomal acidification. In addition to promoting viral degradation, AO-MPs also inhibit the proinflammatory phenotype of AMs, leading to increased treatment efficacy in a SARS-CoV-2-infected mouse model without side effects. These findings highlight the potential use of AO-MPs to treat SARS-CoV-2-infected patients and showcase the feasibility of MP therapies for combatting emerging respiratory viruses in the future.

## Introduction

The global outbreak of novel coronavirus disease 2019 (COVID-19) caused by severe acute respiratory syndrome coronavirus 2 (SARS-CoV-2) has already caused more than 4.5 million deaths worldwide. Despite great efforts in drug development, to date, drugs that can effectively treat SARS-CoV-2 infection are still scarce. While currently available vaccines can greatly prevent viral spread, they are unable to treat infected patients [1]. On the other hand, the screening of small compounds to target steps in the viral life cycle, such as replication and packaging, is facing uncertainty and a lack of safety [2, 3]. Therefore, exploration of unconventional strategies for the treatment of COVID-19 patients is highly desirable and urgently needed.

Advances in material science have provided potential means to target SARS-CoV-2 through effects on the immune system. Cells have the ability to produce extracellular vesicles, including exosomes and microparticles (MPs, also known as microvesicles or ectosomes). Unlike exosomes, which are smaller in size (30–100 nm) and released from endosomes, MPs, which have sizes of 100–1000 nm, are released from the plasma membrane by many cell types in response to stimuli. Previously, we used MPs as nonsynthetic nanocarriers to deliver chemotherapeutic drugs for cancer treatment [4–7]. These drug-packaging MPs not only achieved treatment efficacy against malignant effusions in patients but also showed high treatment safety [6, 7]. Moreover, the preparation of MPs is simple and feasible. In the renin-angiotensin system, angiotensin-converting enzyme 2 (ACE2), a receptor that can be bound by the SARS-CoV-2 surface spike (S) protein and mediate viral entry [8], is known to be widely expressed in tumor cells [9–12], suggesting that ACE2 is likely to be present on the membrane surface of tumor cell MPs. Tumor MPs seem to have certain unique features distinct from those of nontumor cell MPs. For example, the former can polarize macrophages toward the M2 phenotype, but the latter cannot [13]. In addition, although the potential non-self components of tumor MPs readily cause concern about side effects, the use of tumor MPs to treat patients with malignant fluids or cholangiocarcinoma has shown great safety [6, 7, 14, 15]. Based on these analyses, we speculated that tumor MPs might act as a sponge to adsorb SARS-CoV-2 virions and prevent viral spread in vivo. However, even if bound by MPs, the virus is likely to still exist and not disappear entirely, considering that MPs might not have the ability to cleave the virus or cause viral inactivation via a conformational change. Notwithstanding this, virus-carrying MPs can be eliminated by immune cells, especially macrophages performing phagocytosis.

Dry cough is a typical symptom in SARS-CoV-2-infected patients [16], implying that the infection mainly occurs in the lower respiratory tract, especially in the alveoli. The alveoli are air sacs located at the end of the bronchioles, where 90–95% of the resident immune cells are macrophages [17, 18]. Notably, our previous studies have shown that macrophages exhibit highly efficient MP uptake [6, 13, 19]. Moreover, following uptake, MPs are delivered to the lysosomes, a place for cargo degradation by acidic enzymes [13]. Based on these analyses, we hypothesize that T-MPs can act as a sponge-like adsorbent, thus binding up SARS-CoV-2 virions and delivering the virions to the lysosomes in alveolar macrophages (AMs) for degradation.

## Results

### SARS-CoV-2 is adsorbed by ACE2-expressing A549 cell MPs

**Fig. 1:**
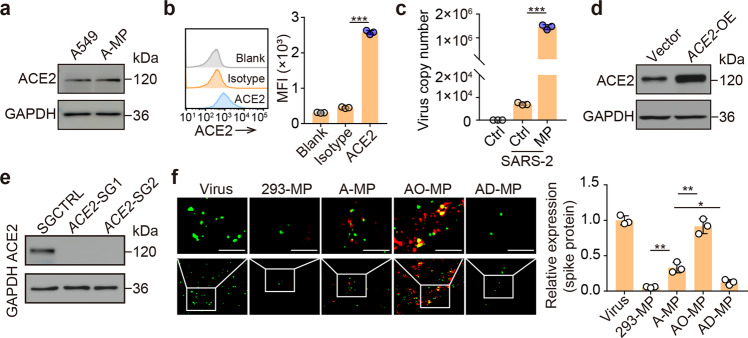
Adsorption of SARS-CoV-2 by ACE2-expressing MPs **a** ACE2 expression in A549 cells and A-MPs was detected by western blotting. **b** Flow cytometry analysis of ACE2 expression in A-MPs. **c** SARS-CoV-2 (5 × 10^4^ TCID_50_) was incubated with 5 × 10^5^ A-MPs (SARS-2/MP) or PBS (SARS-2/Ctrl) for 30 min at 37 °C, and then the mixture or uninfected A-MPs (Ctrl) were filtered through a 0.1-μm filter. The virus load (total virus copy number) on the filter membrane was analyzed by real-time PCR. ACE2 expression in AO-MPs (**d**) and AD-MPs (**e**) was detected by western blotting. **f** MPs (5 × 10^5^) were incubat**e**d with SARS-CoV-2 (5 × 10^4^ TCID_50_) for 30 min at 37 °C. To remove free virus, the MPs were centrifuged, and the pellets were fixed for staining with anti-ACE2 (red) and anti-spike protein (green) antibodies. Representative fluorescence images were obtained under an ultrahigh-resolution structured illumination microscope. Scale bar, 2 μm. The data represent the mean ± SD of three independent experiments. * *p* < 0.05, ** *p* < 0.01, *** *p* < 0.001; one-way ANOVA (**b, f**) or two-tailed Student’s *t* test (**c**)

The A549 cell line is derived from human lung adenocarcinoma tissue and is widely used in animal models [20, 21] and an in vitro assay for type II pulmonary epithelial cells [8, 22]. In our previous studies, we used A549 cell-derived MPs to deliver packaged chemotherapeutic drugs to treat patients with malignant pleural effusion [6, 7]. Significant treatment efficacy was achieved, and no safety concerns were observed [6, 7]. In this study, we hypothesized that A549 cell-derived MPs (A-MPs) could adsorb SARS-CoV-2. Many aspects of A-MPs, including their ~500-nm size and plasma membrane origin, have been characterized in previous studies [7, 23]. The expression of ACE2 in A549 cells has been well reported [12]. In line with this, western blot analysis showed that ACE2 was indeed also expressed on A-MPs (Fig. 1a). Both flow cytometry and immunofluorescence staining confirmed that ACE2 was present on the surface of A-MPs (Fig. 1b and Supplementary Fig. 1c). Next, we determined whether SARS-CoV-2 could be adsorbed by A-MPs. Following the incubation of SARS-CoV-2 with A-MPs, the solutions were filtered through a 0.1-μm filter, which selectively allowed the viral particles to pass but trapped A-MPs on the filter surface. Analysis by real-time PCR showed that viral RNA could be detected in the incubated A-MP samples but not the unincubated A-MP samples (Fig. 1c), suggesting that SARS-CoV-2 is bound by A-MPs. This conclusion was further validated by immunofluorescence staining, which showed the binding of the viral S protein to ACE2 on A-MPs (Supplementary Fig. 1c). To investigate whether A-MPs adsorb SARS-CoV-2 in an ACE2-dependent manner, we additionally prepared MPs from either ACE2-overexpressing A549 tumor cells (AO-MPs) or ACE2-deficient A549 tumor cells (AD-MPs) (Fig. 1d, e and Supplementary Fig. 1a, b, d). We found that AO-MPs carrying more ACE2 molecules could adsorb more SARS-CoV-2 (Fig. 1f and Supplementary Fig. 1e). Intriguingly, AD-MPs still retained approximately 10% capacity to adsorb SARS-CoV-2; however, normal human cell (293T)-derived MPs did not show any ability to adsorb the virus (Fig. 1f), consistent with a previous report [24]. Thus, there must be an ACE2-independent process by which A-MPs inefficiently adsorb SARS-CoV-2. In addition, by quantitating viral numbers, we calculated that every A-MP could adsorb 2.2 viral particles on average and the number increased to 3.5 for AO-MPs (Supplementary Fig. 1e). We thus used AO-MPs for subsequent experiments. Together, these results suggest that SARS-CoV-2 can be effectively adsorbed by A-MPs.

### Adsorbed viruses are delivered into AMs by AO-MPs

**Fig. 2:**
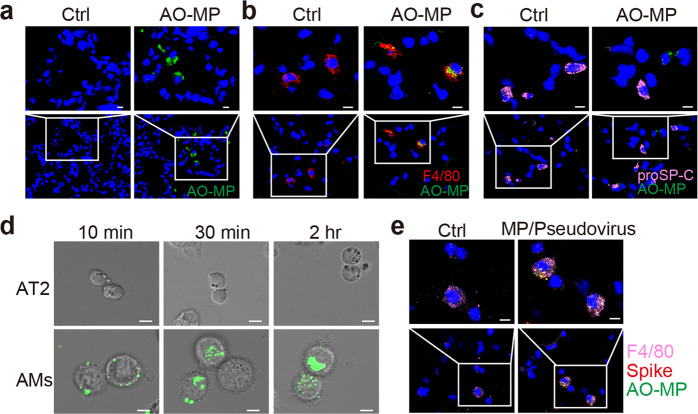
Adsorbed virions are delivered into AMs by AO-MPs. ICR mice were intranasally administered PBS (Ctrl) or PKH67-labeled AO-MPs (green, 5 × 10^6^, 50 μl). Thirty minutes later, lung tissues were collected to prepare frozen sections. The AO-MPs in the lung tissues were observed by confocal microscopy (**a**), and sections were subjected to immunofluorescence staining with anti-F4/80 (red, a marker for macrophages) (**b**) or anti-proSP-C (pink, a marker for type II cells) (**c**) antibodies. a-c, Scale bar, 10 μm. **d** PKH67-labeled AO-MPs (5 × 10^5^) were prepared to adsorb a SARS-CoV-2 pseudovirus at 37 °C for 30 min, and then the virus-adsorbed AO-MPs were incubated with primary AMs and primary alveolar epithelial (AT2) cells isolated from hACE2 transgenic mice for 10 min, 30 min, or 2 h. The uptake of virus-adsorbed AO-MPs by AMs or AT2 cells was analyzed by confocal microscopy. Scale bar, 5 μm. **e** PKH67-labeled AO-MPs (green) were preincubated with a SARS-CoV-2 pseudovirus for 30 min at 37 °C. Then, the mixture or PBS (Ctrl) was intranasally administered to hACE2 transgenic mice. Lung tissues were subjected to immunofluorescence staining with anti-F4/80 (pink) and anti-spike (red) antibodies. Scale bar, 10 μm

Given the clinical symptom of dry cough [16], AO-MPs adsorption of SARS-CoV-2 may occur in the alveoli, and such adsorption will likely not influence viral genomic RNA, which is packaged within the viral envelope. Thus, we asked how AO-MP-adsorbed virions are eliminated in vivo. Following intranasal administration, we observed that AO-MPs were distributed in the alveoli (Fig. 2a). Macrophages are the major immune cell type in the alveoli, where they take up exogenous particles [25]. In line with this, we found that AO-MPs were present in alveolar macrophages (AMs) (Fig. 2b). The alveoli are sac-like structures that are composed of alveolar type I and type II pneumocytes and allow AMs to be located on the surface [26]. We investigated whether AO-MPs were also taken up by alveolar pneumocytes. However, immunostaining did not show colocalization of AO-MPs with pneumocytes (Fig. 2c), suggesting that adsorbed SARS-CoV-2 may not be delivered to pneumocytes by AO-MPs. In addition, an in vitro incubation assay showed that AMs could efficiently take up viral AO-MPs within 10 min; however, isolated primary type II pneumocytes were very inefficient at taking up AO-MPs during the 2-hour incubation (Fig. 2d). This difference in uptake efficiency might be because macrophages are professional phagocytes that are very plastic and readily deform to take up large particles, such as MPs. To further validate this difference in uptake efficiency, we administered SARS-CoV-2 pseudovirus-adsorbed AO-MPs intranasally to mice. As expected, we found that AO-MPs and the virus were colocalized in AMs; in contrast, the presence of AO-MPs and the virus was not observed in pneumocytes (Fig. 2e). Together, these results suggest that AO-MPs can adsorb SARS-CoV-2 viral particles in the alveoli and deliver the virus into AMs.

### AO-MP-delivered SARS-CoV-2 is quenched in AMs

**Fig. 3:**
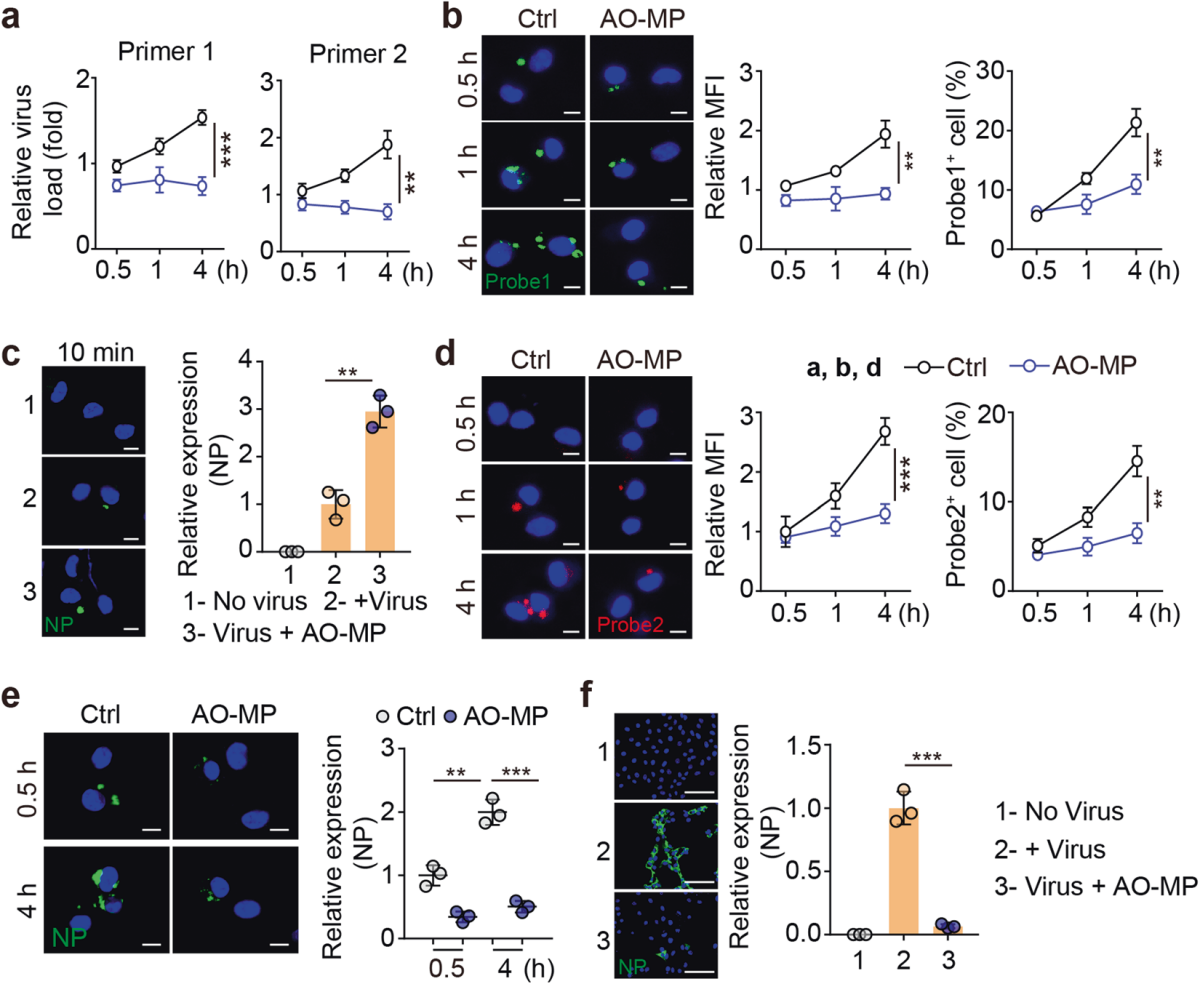
AO-MP-delivered SARS-CoV-2 is quenched in AMs. SARS-CoV-2 (5 × 10^4^ TCID_50_) was incubated with 5 × 10^5^ AO-MPs or PBS (Ctrl) for 30 min at 37 °C and then used to infect AMs. The viral load was determined by real-time PCR with specific primers (**a**), primer1: ORF1ab gene; primer2: N gene, and cells were fixed for RNAscope analysis (**b, d**) or immunohistochemical staining for NP (**e**) at 30 min, 1 h or 4 h post-infection. Probe 1 targeted the viral positive-sense sequence to evaluate the viral distribution (green color) (**a**), and probe 2 targeted the viral negative-sense sequence to investigate viral replication (red color) (**d**). Scale bar, 5 μm. **c** The same as (a), except that cells were fixed for staining with an anti-NP antibody at 10 min post-infection. Scale bar, 5 μm. **f** The same as (a), except that the supernatants were collected at 4 h to infect Vero E6 cells for 48 h. The cells were stained with an anti-NP antibody. Scale bar, 100 μm. The data represent the mean ± SD of three independent experiments. ** *p* < 0.01, *** *p* < 0.001; two tailed Student’s *t* test (**a**–**f**)

Next, we investigated the fate of AO-MP-delivered SARS-CoV-2 in AMs. Previously, we found that SARS-CoV-2 can replicate inside macrophages [27]. Here, we found that an increased viral load in AMs was observed following a 0.5-, 1-, or 4-hr coincubation of SARS-CoV-2 with AMs (Fig. 3a). By performing an RNAscope assay with a green fluorescence-labeled probe against SARS-CoV-2 genomic RNA, we found that increased green spots became apparent as the incubation time increased (Fig. 3b). To our surprise, the use of AO-MP-bound SARS-CoV-2 to treat AMs markedly inhibited the viral load in the cells (Fig. 3a, b). Such contradictory results could not be ascribable to decreased entry of AO-MP-bound SARS-CoV-2 into AMs at the beginning. Instead, in the AO-MP group, more viral particles entered the AMs within the first 10 min of the incubation period (Fig. 3c), suggesting that AO-MPs can efficiently deliver SARS-CoV-2 into AMs but generate an inhibitory effect on viral replication in these cells. Given that SARS-CoV-2 is a positive-sense single-stranded RNA virus, its replication results in the generation of a negative-sense RNA chain [28]. Thus, we used red fluorescence-labeled, negative-sense RNA-specific probes in the RNAScope assay to reflect viral replication. Following a 1-hour incubation with SARS-CoV-2, red spots were present in AMs and were increased with prolonged incubation time (Fig. 3d). However, few red spots were observed in AMs treated with AO-MP-bound SARS-CoV-2 (Fig. 3d). In addition, immunostaining for the NP protein, an essential structural protein involved in the assembly of the SARS-CoV-2 nucleocapsid [29], showed that this viral protein was barely expressed in AO-MP-bound virus-treated AMs but highly expressed in unbound virus-treated AMs (Fig. 3e). In line with this result, supernatants from the AO-MP and unbound virus groups caused weak and strong Vero E6 cell infection, respectively (Fig. 3f). These results suggest that following the delivery of SARS-CoV-2 by AO-MPs, the viral particles can be quenched in AMs.

### AO-MPs prevent viral entry into the cytosol by increasing the endosomal pH

**Fig. 4:**
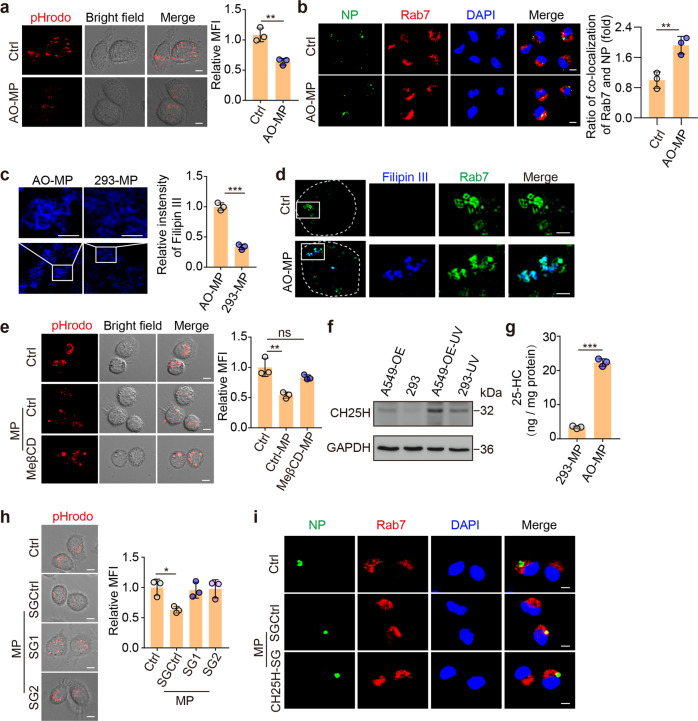
Oxidized cholesterol increases the endosomal pH and disrupts viral entry into the cytosol **a** AMs were pretreated with 5 × 10^5^ AO-MPs for 30 min, stained with pHrodo™ Red Dextran (red), and observed under a confocal microscope. Scale bar, 5 μm. **b** AO-MPs (5 × 10^5^) were incubated with 5 × 10^4^ TCID_50_ SARS-CoV-2 for 30 min at 37 °C and then used to infect AMs for 20 min. The cells were stained with anti-NP and anti-Rab7 (a marker for late endosomes) antibodies. Scale bar, 5 μm. **c** MPs were stained with filipin III and observed by ultrahigh-resolution structured illumination microscopy. Scale bar, 1 μm. **d** Filipin III-labeled MPs (5 × 10^5^) were incubated with AMs for 20 min, and then the cells were stained with anti-Rab7 antibodies. Representative cells are shown outlined by dashed white lines, and the boxed areas are magnified on the right. Scale bar, 1 μm. **e** The same as (**a**), except MPs generated from ACE2-overexpressing A549 cells (A549-OE) or MeβCD-treated A549-OE cells were used. Scale bar, 5 μm. **f** CH25H expression was detected by western blotting after UV irradiation. **g** The 25-HC levels in MPs were quantified by LC–MS/MS. **h, i**, The same as (**a**) and (**b**), respectively, except MPs were generated from A549-OE cells or CH25H-knockout cells. SG1 and SG2 are guide RNAs for knocking out *CH25H*. Scale bar, 5 μm. The data represent the mean ± SD of three independent experiments. ^ns^ no statistical significance, * *p* < 0.05, ** *p* < 0.01, *** *p* < 0.001; two-tailed Student’s *t* test (**a**–**c**, **g**) or one-way ANOVA (**e, h**)

Next, we investigated how AO-MP-bound viruses were quenched in AMs. Macrophages use the phagocytotic pathway to take up viral particles. In this way, SARS-CoV-2 would be transferred from the extracellular space to the endosomes and further to the lysosomes [30], where large amounts of enzymes are present and ready to degrade the virus in a low pH-dependent manner [31, 32]. Biologically, viruses may evolve mechanisms to evade this degradation. SARS-CoV-2 can exploit endosomal acidification for the cleavage of its surface spike protein, leading to viral envelope fusion with the endocytic membrane and release of viral genomic RNA into the cytoplasm to initiate viral replication [8, 33]. pHrodo™ Red Dextran is an acid-sensitive fluorescent dye that can be used to measure the endosomal pH [34]. We found that attenuated dextran fluorescence was present in the endosomes of AO-MP-treated AMs compared to those of untreated AMs (Fig. 4a). In line with this result, the viral NP protein was colocalized with Rab7, a late endosomal marker, in treated AMs but not in untreated AMs (Fig. 4b), suggesting that AO-MP delivery prevents SARS-CoV-2 escape from the endosomes. Intriguingly, in contrast to AO-MPs, 293T cell-derived MPs did not increase the endosomal pH of AMs (Supplementary Fig. 2a) and could not maintain viral retention in the endosomes (Supplementary Fig. 2b). In addition, the use of AD-MPs led to a similar increase in the endosomal pH as AO-MP use did (Supplementary Fig. 2c). These results suggest that AO-MPs can inhibit the acidification of endosomes and hinder viral entry into the cytoplasm.

### Oxidized cholesterol in AO-MPs interferes with endosomal proton pumps

Next, we explored the manner by which phagocytosed AO-MPs caused the increase in the endosomal pH. Following endocytosis, vacuolar H^+^-ATPase (v-ATPase) is recruited to the endosomal membrane, which pumps H^+^ from the cytosol into the endosomal lumen to generate an acidic pH [35]. v-ATPase is a protein complex assembled in lipid rafts [36, 37]. Given the high enrichment of lipid rafts and cholesterol in the MP membrane [4], we assumed that MP membrane-contained cholesterol could somehow be sorted to the endosomal membrane, thus regulating proton pump formation and/or function. We found that AO-MPs had much more cholesterol than 293-MPs (Fig. 4c and Supplementary Fig. 2d). Through imaging filipin III-labeled cholesterol by high-resolution confocal imaging, we observed the translocation of cholesterol from AO-MPs into the endosomal membrane (Fig. 4d). MeβCD is a clinically used drug that is able to extract cholesterol from the plasma membrane [38]. We thus prepared MPs from MeβCD-treated ACE2-overexpressing A549 cells (Supplementary Fig. 2e, f). Indeed, the treated MPs with a lower cholesterol content were found to lack the ability to increase the endosomal pH (Fig. 4e). Liposomes are synthesized nanoparticles that are mainly composed of cholesterol [39]. However, we found that liposomes did not increase the endosomal pH of macrophages (Supplementary Fig. 2g). This inconsistency prompted us to further explore the possible difference in cholesterol between AO-MPs and liposomes. A549 cells were irradiated with ultraviolet light to increase the production of MPs [40], but no irradiation was performed during liposome preparation [41]. Notably, irradiation may generate strong oxidative stress [42, 43]. Here, we wondered whether oxidative stress could alter the structure of cholesterol derivatives, such as oxysterol. Cholesterol 25-hydroxylase (CH25H) oxidizes cholesterol to 25-hydroxycholesterol (25-HC). Intriguingly, we found that UV irradiation resulted in higher expression of CH25H in ACE2-overexpressing A549 cells than in 293T cells (Fig. 4f and Supplementary Fig. 2h). LC-MS/MS analysis showed that 25-HC was highly present in AO-MPs, which exhibited a 7-fold increase compared to irradiated 293T cells (Fig. 4g). Then, we knocked out CH25H to block cholesterol oxidation (Supplementary Fig. 2i). As a result, AO-MPs without oxidized cholesterol did not increase the endosomal pH (Fig. 4h). Furthermore, these MPs could not retain the virus in the endosomes of macrophages (Fig. 4i). When we used 25-HC to treat AMs, we found that compared to the control, 25-HC had the ability to increase the endosomal pH directly (Supplementary Fig. 2j). Together, these results suggest that the increase in the endosomal pH induced by AO-MPs is mediated by oxidized cholesterol.

### AO-MPs facilitate viral clearance by decreasing the lysosomal pH

**Fig. 5:**
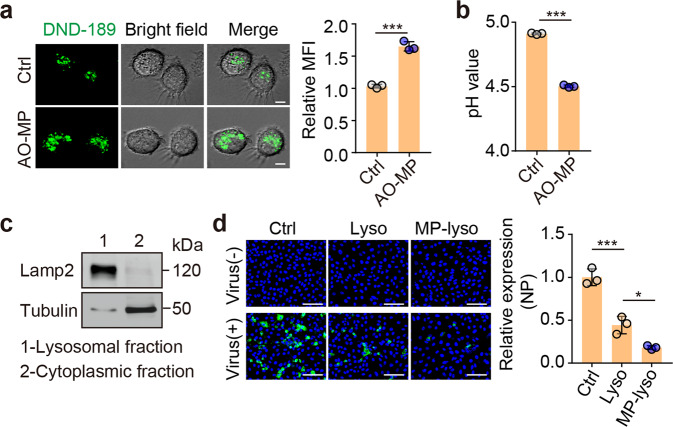
AO-MPs facilitate viral clearance by decreasing the lysosomal pH **a** AMs were pretreated with AO-MPs for 30 min and then stained with LysoSensor™ Green DND-189 for 30 min at 37 °C. The cells were observed under a confocal microscope. **b** The same as (**a**), except that cells were stained with LysoSensor™ Yellow/Blue DND-160. The pH value was detected by a microplate reader. **c** Cells were processed to separate the lysosomal and cytoplasmic fractions of AO-MP-treated or untreated RAW264.7 cells. The purified lysosomes were detected by western blotting. **d** The lysates of purified lysosomes isolated from AO-MP-treated (MP-lyso) or untreated (Lyso) RAW264.7 cells were incubated with SARS-CoV-2 for 30 min at 37 °C and then used to infect Vero E6 cells for 48 h. Cells were stained with an anti-NP antibody. Lyso, lysosomes. Scale bar, 50 μm. The data represent the mean ± SD of three independent experiments. * *p* < 0.05, *** *p* < 0.001; two-tailed Student’s *t-*test (**a, b**) or one-way ANOVA (**d**)

Interfering with the pumping of protons from the cytosol into the endosomal lumen may leave more protons in the cytosol. To maintain a stable cytosolic pH, v-ATPase probably pumps protons into the Golgi and lysosomes [44, 45]. We found that endosome-detained SARS-CoV-2 could be escorted to the lysosomes (Supplementary Fig. 3a), in which the virus may be degraded by lysosomal enzymes in an acidic pH-dependent manner [31]. Lysosomal v-ATPase is a sensor of the cytosolic pH [46]. When we used dimethyl amiloride (DMA), a sodium-hydrogen exchanger inhibitor that can increase the level of cytosolic hydrogen ions in macrophages [47], to treat AMs, we observed a decrease in the lysosomal pH (Supplementary Fig. 3b), implying that oxidized cholesterol might favor the pumping of protons into the lysosomal lumen by impeding the entry of protons into the endosomes. Indeed, in contrast to the increased endosomal pH, the lysosomal pH was decreased in AO-MP-treated AMs (Fig. 5a, b), consistent with a previous report [27]. However, the use of normal cell-derived MPs or liposomes to treat macrophages did not alter the lysosomal pH (Supplementary Fig. 3c, d). In line with the decreased lysosomal pH, we found that the lysates of lysosomes isolated from AO-MP-treated RAW264.7 macrophages could more efficiently inactivate SARS-CoV-2 than those from control counterparts (Fig. 5c, d, and Supplementary Fig. 3e). Together, these results suggest that AO-MPs are able to facilitate the lysosomal degradation of SARS-CoV-2 by enhancing lysosomal acidity.

### Virus-adsorbed AO-MPs polarize AMs toward an anti-inflammatory phenotype

**Fig. 6:**
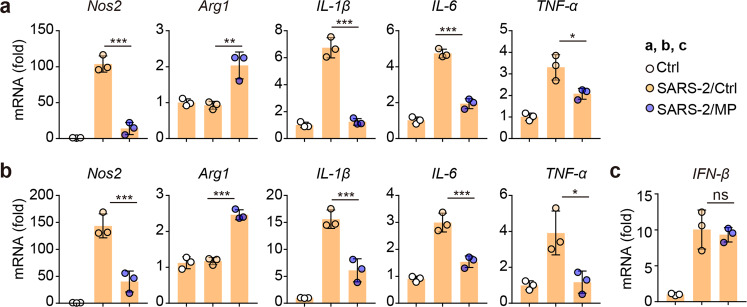
Virus-adsorbed AO-MPs polarize AMs toward the M2 phenotype **a** AMs were infected with 5 × 10^4^ TCID_50_ SARS-CoV-2 for 24 h. The mRNA levels of *Nos2, Arg1, IL-1β*, *IL-6*, and *TNF-α* were detected by qPCR. **b, c** SARS-CoV-2 (1 × 10^5^ TCID_50_) was incubated with 5 × 10^6^ AO-MPs (SARS-2/MP) or PBS (SARS-2/Ctrl) for 30 min at 37 °C, and then the mixture or PBS (Ctrl) was intranasally administered to hACE2-transgenic mice (*n* = 3). After 24 h, the relative gene expression in AMs was detected by qPCR. The data represent the mean ± SD of three independent experiments. ^ns^ no statistical significance, * *p* < 0.05, ** *p* < 0.01, *** *p* < 0.001; one-way ANOVA (**a**–**c**)

Virus-infected macrophages commonly upregulate proinflammatory cytokines, which are thought to exacerbate the pathogenesis of SARS-CoV-2 infection. The upregulation of a panel of proinflammatory factors in SARS-CoV-2-infected AMs has been reported [48, 49]; on the other hand, A-MPs are able to induce macrophages to release anti-inflammatory cytokines [13], prompting us to test whether SARS-CoV-2-adsorbed AO-MPs polarize macrophages toward an anti-inflammatory phenotype. Again, we found that single SARS-CoV-2 infection stimulated macrophages to upregulate TNF-α, IL-1β, IL-6, and inducible nitric oxide synthase (iNOS); however, SARS-CoV-2-adsorbed AO-MP treatment of macrophages resulted in upregulation of the expression of arginase 1 but downregulation of that of the proinflammatory factors (Fig. 6a). To validate this result in vivo, we either infected hACE2-transgenic mice with SARS-CoV-2 or treated the mice with SARS-CoV-2-adsorbed AO-MPs intranasally. Through this comparison, we found that isolated AMs exhibited either a proinflammatory phenotype or an anti-inflammatory phenotype (Fig. 6b). Notably, SARS-CoV-2-adsorbed AO-MPs did not alter the SARS-CoV-2-induced expression of type I interferons in AMs (Fig. 6c). Together, these results suggest that the delivery of SARS-CoV-2 to AMs by AO-MPs can avoid the induction of inflammation.

### AO-MPs are a therapeutic agent to treat SARS-CoV-2 infection in vivo

**Fig. 7:**
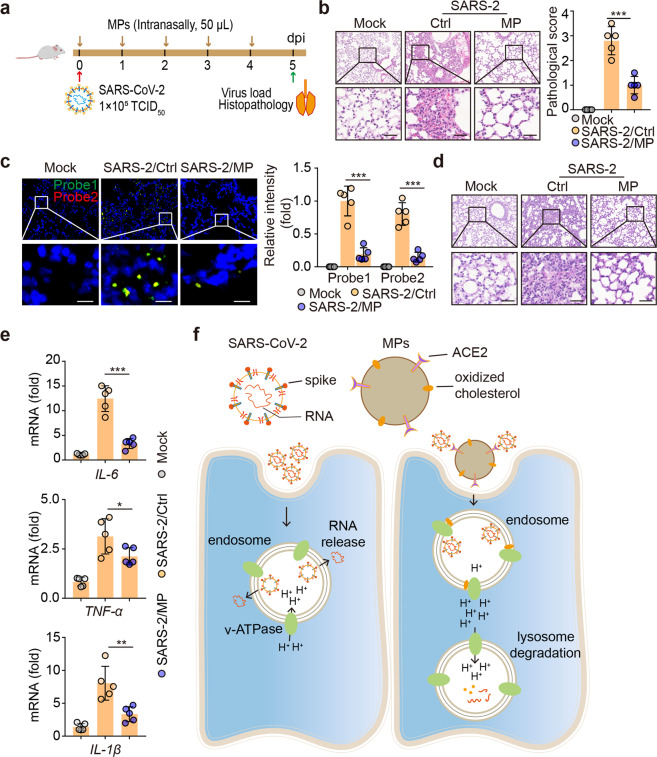
Treatment of SARS-CoV-2 infection with AO-MPs in vivo. Schematic diagram of the experimental design. hACE2-transgenic mice were infected with 1 × 10^5^ TCID_50_ SARS-CoV-2 and then administered AO-MPs (i.n., 50 μl, 5 × 10^6^) once per day for 5 days (**a**). The control group (Ctrl) received the vehicle (PBS) as a placebo. Lung tissues were fixed for H&E staining (**b**, *n* = 5), RNAscope analysis with probes 1 (green) and 2 (red) (**c**, *n* = 5), and PAS staining (**d**, *n* = 5). Three lung sections from the left lobe were evaluated for each mouse. The representative images selected reflect the distributions of damaged lung tissues. Scale bar, 50 μm for **b** and **d**, 10 μm for **c**. **e**, The mRNA levels of *IL-1β*, *IL-6* and *TNF-α* in lung tissues were detected by qPCR. **f** Schematic of AO-MP-mediated SARS-CoV-2 degradation in AMs. The data represent mean ± SD. * *p* < 0.05, ** *p* < 0.01, *** *p* < 0.001; two-tailed Student’s *t-*test (**b, c**) or one-way ANOVA (**e**)

Finally, we explored the potential use of ACE2-overexpressing AO-MPs to treat SARS-CoV-2 infection. Using hACE2-transgenic mice as a model, we infected the mice with SARS-CoV-2, followed by treatment with intranasal AO-MPs once per day for 5 days (Fig. 7a). This daily treatment was based on the observation that MPs administered intranasally were cleared 24 h later (Supplementary Fig. 4a). H&E staining showed less peribronchial and perivascular inflammatory cell infiltration and reduced pathological damage in the lungs of the treated mice (Fig. 7b). In line with this morphological amelioration, a decreased viral load in the lungs was demonstrated by an RNAscope assay (Fig. 7c), immunostaining for the NP protein and real-time PCR (Supplementary Fig. 4b, c). Previously, we found that alveolar mucus is pathologically produced during SARS-CoV-2 infection [50]. Here, we also observed that the amount of mucus in the lungs was reduced in the AO-MP-treated mice (Fig. 7d and Supplementary Fig. 4d). In addition, we did not observe any side effects of AO-MP treatment. Alanine transaminase (ALT), aspartate transaminase (AST), creatine, and mouse weight were not altered by AO-MP treatment (Supplementary Fig. 4e, f). Again, in the treated lung tissues, lower levels of proinflammatory factors were detected (Fig. 7e). In addition, we infected mice with SARS-CoV-2 2 h before AO-MP intranasal treatment to properly assess the therapeutic efficacy of AO-MPs in a realistic state. Consistent results were observed, with the mice exhibiting a decreased viral load, reduced lung pathology, and decreased expression of IL-6 and TNF-α (Supplementary Fig. 4g–i). Furthermore, as a comparison, we used AD-MPs to treat pre-infected mice. We found that although AD-MPs moderately lowered the viral load, their effect was much weaker than that of AO-MPs, concomitant with a worse lung pathology. Nevertheless, the effect of AD-MPs on IL-6 and TNF-α mRNA expression exhibited only a slight increase compared to that of AO-MPs (Supplementary Fig. 4g–i). Together, these results suggest that AO-MP treatment inhibits SARS-CoV-2 infection and ameliorates lung pathology.

## Discussion

SARS-CoV-2 invades the respiratory tract and causes pathogenesis. Anatomically, the respiratory trachea branches off into two bronchi, and the bronchi are further divided into bronchioles and respiratory bronchioles, which end in alveoli [51]. The bronchi and bronchioles are mainly composed of ciliated epithelial cells and mucin-producing goblet cells, with the cilia sweeping off particle-trapping mucus [52]. Thus, a virus invading the bronchial or bronchiolar epithelium readily causes cough with sputum. The clinical symptom of dry cough in SARS-CoV-2-infected patients suggests that this virus mainly invades the alveoli rather than the bronchioles or bronchi. The alveolus is a tiny, thin-walled, capillary-rich sac structure where alveolar macrophages reside to clear billions of inhaled particles, allergens, and microbes daily [53]. Cellular microparticles are vesicular plasma membrane fragments with a diameter of 0.1~1 μm that are shed by cells in response to various physiological and artificial stimuli [4]. In this study, we provide evidence that AO-MPs administered intranasally can access the alveoli, where the MPs adsorb SARS-CoV-2 and deliver the virus to AMs, leading to viral clearance by the AMs.

Tumor cell MPs can be versatile carriers and show unique advantages. First, tumor cells can be easily expanded in vitro, thus generating a large number of MPs through a simple process; second, MPs are formed from cellular membranes, making them much safer and less likely to be rejected; and third, MPs can package or carry different kinds of materials, including viruses [23]. In this study, we further show that AO-MPs can use expressed ACE2 to adsorb SARS-CoV-2 on their surface. In the respiratory tract, goblet cells release mucus, which may adhere to cilia and form a mesh-like structure via cilia movement [54]. Such a reticular structure may exert a sticking function to prevent exogenous particles from entering the alveoli. However, cellular MPs seem to have the ability to escape this trap and enter the alveoli, where they interact with and adsorb SARS-CoV-2, followed by AM uptake. Therefore, this study promises a simple and innocuous therapeutic intervention for treating SARS-CoV-2 infection. Notwithstanding this, intrinsic parameters may impact the ability of MPs to exert their function. For instance, MPs with a larger size and higher ACE2 density may be more efficient in capturing SARS-CoV-2 virions. However, whether a large size affects the uptake efficiency of macrophages remains unclear. Thus, exploration to determine the suitable size is valuable for optimizing the MP-based approach against SARS-CoV-2 infection.

In the alveoli, following uptake by AMs, AO-MPs can effectively escort SARS-CoV-2 from the endosomes to the lysosomes for degradation by regulating the endolysosomal pH. Endosome maturation from the early to late stage involves a gradual acidification process [35]. However, this acidification favors SARS-CoV-2 escape from the endosome and entry into the cytosol because a low pH is required for the cleavage of the spike protein, thus allowing viral envelope fusion with the endosomal membrane and resulting in the release of viral RNA into the cytoplasm [8]. In this study, we found that AO-MPs interfered with endosomal acidification by targeting endosomal lipid rafts, a structure required for v-ATPase to pump protons. This interference was attributed to the oxidized cholesterol in AO-MPs. Under normal conditions, the cellular membrane usually contains a very low level of oxidized cholesterol [55]. However, under oxidative stress, cells may upregulate 25-hydroxylase, which catalyzes cholesterol oxidation. The use of this mechanism by cells, especially tumor cells, seems to avoid attack by free radicals on important intracellular biomolecules. During the preparation of AO-MPs, UV irradiation exposes tumor cells to high-energy waves, leading to electron transfer, the generation of abundant free radicals, and the induction of 25-hydroxylase. As a result, oxidized cholesterol is enriched in prepared AO-MPs. However, in macrophage endosomes, this oxidized cholesterol can be translocated from AO-MPs to the endosomal lipid rafts, thus impairing the pumping of H^+^ into the endosomal lumen. Notably, an increase in pH prevents viral escape; however, a decrease in pH is required for lysosomal degradation of the virus. This is because the activity of lysosomal enzymes relies on a low pH [31]. In this study, we also found that AO-MPs could decrease the lysosomal pH, thus facilitating viral clearance. Although we provide some evidence that this decrease in the lysosomal pH might be due to the maintenance of cytosolic H^+^ homeostasis, the detailed mechanism needs to be further investigated.

Viral particle clearance is undoubtedly crucial in the treatment of SARS-CoV-2-infected patients. However, inhibition of inflammation is also important in hindering viral infection-induced pathogenesis. During SARS-CoV-2 infection, activated macrophages may release a large amount of proinflammatory cytokines, which may even cause lethal cytokine release syndrome [49]. By analyzing SARS-CoV-2-infected AMs both in vitro and in vivo, we consistently found that AMs could be activated by SARS-CoV-2 to upregulate the expression of proinflammatory cytokines and antiviral type I interferons. However, while AO-MP-delivered SARS-CoV-2 did not induce proinflammatory cytokines, it still increased type I interferons, suggesting that the anti-inflammatory and antiviral responses of AMs can be separated. Tumor cell-derived MPs are capable of polarizing macrophages toward the M2 phenotype [13], which may explain the above anti-inflammatory effects. On the other hand, tumor MPs contain fragments of tumor cell mitochondrial and genomic DNA, which may activate the cGAS-STING pathway for type I interferon induction [13, 56]. The separation of anti-inflammatory and antiviral responses has also been observed in manganese salt-treated macrophages [57]. Thus, AO-MPs therapeutically promote viral clearance through the endolysosomal system; moreover, AO-MPs inhibit inflammatory innate immunity but enhance antiviral interferon-mediated immunity.

In summary, the data in this study show that AO-MPs, by virtue of their capacity to adsorb SARS-CoV-2 and regulate the endolysosomal pH and inflammatory activity of AMs, can act as a therapeutic agent against SARS-CoV-2 infection. AO-MPs effectively access the alveoli, where they adsorb and deliver the virus into the endosomes and subsequently the lysosomes of AMs for degradation (Fig. 7f). AO-MPs may also directly neutralize SARS-CoV-2, thus reducing viral infection of alveolar epithelial cells. In addition, virus-containing AMs may be passively expelled to the upper mucocilliary epithelium by the exhalation force. Our approach is different from the previously reported decoy nanoparticles and nanosponges, which also trap SARS-CoV-2:[58, 59] the differences lie in (1) preparation, as we use cell death to naturally form MPs rather than artificially producing decoy nanoparticles or nanosponges; (2) our MPs contain oxidized cholesterol; and (3) our MPs do not technically adsorb proinflammatory cytokines but induce macrophages to become anti-inflammatory cells. Overall, AO-MPs, as a natural biomaterial, can function as an accelerator of viral clearance, leading to the treatment of SARS-CoV-2 infection with high efficacy and safety.

## Materials and methods

### Animals and cell lines

Female hACE2-transgenic ICR mice, 6–8 weeks old, were purchased from the Center of Medical Experimental Animals of the Chinese Academy of Medical Sciences (Beijing, China). The murine macrophage cell line RAW264.7, human alveolar basal epithelial carcinoma cell line A549, and African green monkey kidney cell line Vero E6 were purchased from the Cell Resource Centre of Peking Union Medical College (Beijing, China) and cultured in DMEM (Gibco, USA) supplemented with 10% FBS.

### Preparation of MPs

A549, *ACE2*-overexpressing A549, *ACE2*-deficient A549 and 293T cells were exposed to ultraviolet radiation (300 J/m^2^, UVC) for 1.5 h, and 18 h later, the supernatants were collected to isolate microparticles as described previously [4]. Briefly, the supernatants were centrifuged at 1000 × *g* for 10 min to remove cells and then centrifuged at 14,000 × *g* for 2 min to remove debris. Afterward, the supernatants were centrifuged at 14,000 × *g* for 60 min at 4 °C to pellet MPs. The MPs were washed three times and suspended in PBS for subsequent experiments. The number of MPs was calculated by flow cytometry.

### MP labeling

All the steps were performed according to the manufacturer’s protocol (PKH67 Fluorescent Cell Linker Kits, Thermo Fisher Scientific, Cat: PKH67GL). Briefly, MPs washed with PBS were centrifuged at 14,000 × *g* for 30 min. The pellet was suspended in 1 mL of Diluent C with 2 μL of PKH67 dye solution at 37 °C for 5 min. Then, the reaction was stopped by adding an equal volume of serum and centrifuged at 14,000 × *g* and 4 °C for 30 min. The MPs were washed twice with PBS and prepared for subsequent experiments. For imaging by ultrahigh-resolution structured illumination microscopy, labeled MPs were fixed with 4% paraformaldehyde in the dark and centrifuged at 1500 × *g* for 3 min with Cytospin 4 (Thermo Fisher Scientific).

### Generation of a knockout cell line with CRISPR–Cas9

For construction of the stable *ACE2*-knockout cell line, the following sgRNAs targeting *ACE2* were used: SGCTRL,GGGCGAGGAGCTGTTCACCG (sense) and CGGTGAACAGCTCCTCGCCCC (antisense); *ACE2*-SGRNA1, TATGTGCACA

AAGGTGACAA (sense) and TTGTCACCTTTGTGCACATA (antisense); and *ACE2*-SGRNA2, TGACAGCTCATCATGAGATG (sense) and CATCTCATGATGAGCTG

TCA (antisense). For construction of the stable *CH25H*-knockout cell line, the following sgRNAs targeting *CH25H* were used: *CH25H*-SGRNA1, CTGGGACCACCTGAGGAGCT (sense) and AGCTCCTCAGGTGGTCCCAG (antisense); and *CH25H*-SGRNA2, AGCCCCTCTGGGACCACCTG (sense) and CAGGTGGTCCCAGAGGGGCT (antisense). These sgRNAs were cloned into the pSpCas9(BB)-2A-GFP vector plasmid (Addgene, Cat. 48138) and transfected into cells. Forty-eight hours later, GFP-positive cells were sorted by flow cytometry using a BD Biosciences FACSAria III. The candidate knockout cells were verified by western blotting or immunofluorescence staining.

### Stable overexpression of ACE2 in the A549 cell line

The human ACE2 coding sequence was amplified and inserted into the amphotropic vector plasmid pLV-EF1α-IRES-Puro (Addgene, Cat. 85132) for transient expression in 293T cells to obtain virus containing the target gene. A549 cells transduced with lentiviruses containing hACE2 were selected with 1 μg/ml puromycin to obtain ACE2-overexpressing A549 cell clones.

### Lysosome isolation

All the steps were performed according to the manufacturer’s protocol at 4 °C (Lysosome Extraction Kit, Sigma, LYSISO1). Briefly, adherent cells were trypsinized and washed with cold PBS. The cell pellet was resuspended and lysed in a 7-ml Dounce homogenizer. Then, the cell homogenates were centrifuged for 10 min at 1000 × *g*. Subsequently, the supernatants were centrifuged for 20 min at 20,000 × *g* to pellet the lysosomes and other organelles. Following density gradient centrifugation for 4 h at 150,000 × *g* in an SW50.1 rotor, the highest (least dense) band was removed and diluted in PBS. The lysosomes were washed and pelleted by centrifugation at 20,000 × *g* for 20 min. Finally, the precipitate was analyzed by western blotting or immunofluorescence staining.

### Isolation of primary alveolar macrophages and alveolar epithelial type II cells

Primary alveolar macrophages (AMs) were isolated from murine bronchoalveolar lavage fluid (BALF). Briefly, mice were anesthetized immediately prior to lavage, and the trachea was dissected. The lungs were lavaged five times with 1 ml of PBS, and the retained BALF was centrifuged at 600 × *g* and 4 °C for 5 min. The pellet was harvested, resuspended in complete RPMI 1640 medium, and then incubated in a culture plate for 2 hr. Then, the nonadherent cells were removed by gentle washing with PBS. Primary alveolar epithelial (AT2) cells were isolated from hACE2 mice as previously reported [60]. Briefly, mice were perfused with 10 ml of cold PBS through the right ventricle. The lungs were filled with 2 ml of dispase (BD Bioscience, USA) and low-gelling-temperature agarose (Sigma Aldrich, USA) before the lung tissues were incubated with 2 ml of dispase at 37 °C for 20 min. Then, the lung tissues were mechanically dissociated, and the slurry was filtered through 70- and 40-μm nylon meshes (JETBIOFIL, China). The cellular suspension was incubated with biotinylated anti-CD45 (BioLegend, clone 30-F11, Cat. 103104), anti-CD16/32 (BD Pharmingen™, clone 2.4G2, Cat. 553143), anti-CD31 (BioLegend, clone MEC13.3, Cat. 102504), anti-TER119 (BioLegend, clone TER119, Cat. 116104) and anti-CD104 (BioLegend, clone 346–11A, Cat. 12603) antibodies at 4 °C for 30 min, and then Dynabeads® MyOne^TM^ streptavidin T1 magnetic beads (Thermo Fisher Scientific, Cat. 65601) were added to the cell suspension to exclude leukocytes, monocytes/macrophages, NK cells, neutrophils, endothelial cells, and erythroid cells. Negative selection of fibroblasts was performed by adherence to noncoated plastic plates. Cell purity was assessed routinely by flow cytometry.

### Flow cytometry

A-MPs were resuspended in PBS with 2% FBS containing an anti-ACE2 antibody (Gene Tex, Cat. GTX101395, 1:200) and incubated for 30 min. The MPs were washed and stained with a goat anti-rabbit antibody (Thermo Fisher Scientific, Cat. A-11034) for 30 min. Data were acquired using an Accuri C6 system (BD Biosciences) and analyzed with FlowJo software.

### Immunofluorescence staining

Cells were fixed in 4% paraformaldehyde and permeabilized with 0.2% Triton X-100. The fixed cells were blocked in 5% BSA and incubated with an anti-Lamp2 (Abcam, Cat. ab25339, 1:200), anti-SARS spike protein (Abcam, Cat. Ab273433, 1:200), anti-SARS nucleocapsid protein (Abcam, Cat. Ab273434, 1:200), anti-Rab7 (Abcam, Cat. ab137029, 1:200), anti-Rab5 (CST, Cat. 3547 S) or anti-ACE2 (Gene Tex, Cat. GTX101395, 1:200) antibody at 4 °C overnight, and then the cells were washed and incubated with secondary antibodies for 1 h at room temperature. Finally, the slides were counterstained with DAPI and mounted for confocal analysis. The intensity of the immunofluorescence staining was analyzed with ImageJ 9.0 software.

### Histological and immunohistochemical staining

Lung tissues from mice were fixed in 10% formalin, embedded in paraffin, and sectioned for H&E staining. According to the morphological changes observed after SARS-CoV-2 infection, the lung tissues were graded as mild (1), moderate (2), severe (3), or life-threatening (4). An expert in pathology who was blinded to the experiment scored the sections based on inflammatory cell infiltration, parenchymal pneumonia, alveolar hemorrhage, and bronchiolar/bronchial luminal or alveolar exudate. Immunohistochemical staining was performed according to a protocol described previously [61]. In brief, sections of paraffin-embedded tissues were incubated with an anti-mucin 1 (1:200, Abcam, Cat. ab45167), anti-mucin 5a (1:200, Abcam, Cat. ab24071), anti-mucin 5b (1:200, Abcam, Cat. ab77995) or anti-SARS nucleocapsid protein (1:500, Abcam, Cat. ab273434) antibody at 4 °C overnight. Afterward, the slides were sequentially incubated with two HRP-conjugated secondary antibodies for 1 h at room temperature. The slides were incubated with ANO Reagent PPD520 or PPD570 using a PANO 4-plex IHC Kit (Panovue, China) according to the manufacturer’s instructions, followed by counterstaining with DAPI (Thermo, USA) and finally mounting for analysis. Immunohistochemical staining was also conducted on 8-μm frozen sections. An anti-F4/80 (1:200, Abcam, Cat. ab6640) or anti-Prosurfactant Protein C (1:500, Abcam, Cat. Ab211326) antibody was used. The stained lung sections were scanned and digitalized utilizing a TissueFaxs Plus System coupled to a Zeiss Axio Imager Z2 microscope or Nikon A1 confocal microscope. The intensity of positive staining was analyzed with ImageJ 9.0 software.

### Real-time PCR

Total RNA was extracted from cells or viruses using TRIzol (Invitrogen) and reverse transcribed into cDNA by using a high-capacity cDNA reverse transcription kit (Applied Biosystems, CA). The primer sequences were as follows: *Gapdh*, 5′-AGGTCGGTGTGAACGGATTTG-3′ (sense) and 5′-TGTAGACCATGTAG

TTGAGGTCA-3′ (antisense); SARS-CoV-2 primer1 (*ORF1ab*): 5′-CCCTGTG

GGTTTTACACTTAA-3′ (sense) and 5′-ACGATTGTGCATCAGCTGA-3′ (antisense); SARS-CoV-2 primer2 (*N*): 5′-GGGGAACTTCTCCTGCTAGAAT-3′ (sense) and 5′-CAGACATTTTGCTCTCAAGCTG-3′ (antisense); *Nos2*, 5′-GATGTTGAACTA

TGTCCTATCTCC-3′ (sense) and 5′-GAACACCACTTTCACCAAGAC-3′ (antisense); *Arg1*, 5′-CAAGACAGGGCTCCTTTCAG-3′ (sense) and 5′-TGGCTTAT

GGTTACCCTCCC-3′ (antisense); *TNF-α*, 5′-CCACGTCGTAGCAAACCAC-3′ (sense) and 5′-TTGTCCCTTGAAGAGAACCTG-3′ (antisense); *IL-1β*, 5′-GCAACTGTTCCTGAACTCAACT -3′ (sense) and 5′-ATCTTTTGGGGTCCGTCA

ACT-3′ (antisense); *IL-6*, 5′-TAGTCCTTCCTACCCCAATTTCC-3′ (sense) and 5′-TTGGTCCTTAGCCACTCCTTC-3′ (antisense); *CH25H*, 5′-GCTGGCAACGC

AGTATATGAG-3′ (sense) and 5′-CGAGCAGTGTGACGTTCATC-3′ (antisense); and *IFN-β*, 5′-CAGCTCCAAGAAAGGACGAAC-3′ (sense) and 5′-GGCAGTGTAACTCTTCTGCAT-3′ (antisense). To quantify the SARS-CoV-2 N gene copy number, SARS-CoV-2-N-probe (5′-FAM- TTGCTGCTGCTTGACAGATT-TAMRA-3′) was used as described previously [62]. Real-time PCR was performed using ABI QuantStudio 3 (Applied Biosystems, CA, USA). Data are reported as the mean ± SD of three independent experiments performed in duplicate.

### RNA in situ hybridization (RNA-ISH)

RNA-ISH was performed on primary alveolar macrophages grown on glass coverslips or paraffin-embedded 5-μm lung tissue sections using the RNAscope Multiplex Fluorescent Assay v2 according to the manufacturer’s instructions (Advanced Cell Diagnostics, USA). Briefly, cells were fixed in 4% paraformaldehyde and incubated with hydrogen peroxide at RT for 10 min and 1:15 diluted Protease III at RT for 10 min. Lung tissue sections were deparaffinized with xylene, rehydrated with graded ethanol, incubated with hydrogen peroxide, and then boiled for 15 min in Target Retrieval buffer, followed by incubation with Protease Plus for 15 min at 40 °C. The slides were hybridized with SARS-CoV-2 probes in a hybridization oven at 40 °C for 2 h, and the fluorescence signals were amplified according to the manufacturer’s protocol. The cells grown on glass coverslips and stained lung sections were scanned and digitalized utilizing a TissueFaxs Plus System coupled to a Zeiss Axio Imager Z2 microscope. The fluorescence intensity and positive cell rate were analyzed with ImageJ 9.0 software.

### Lysosomal pH measurement

LysoSensor^TM^ Yellow/Blue DND-160 (Thermo Fisher, USA), which exhibits a pH-dependent dual-excitation spectrum in living cells, was utilized to quantify the pH of macrophage lysosomes according to the manufacturer’s guidelines. LysoSensor^TM^ Yellow/Blue DND-160 emits predominantly yellow fluorescence in acidic environments and blue fluorescence in alkaline environments. In brief, cells were treated with 5 μM LysoSensor^TM^ Yellow/Blue DND-160 in prewarmed medium for 5 min at 37 °C. After washing twice with cold PBS, the labeled cells were incubated at 37 °C for 5 min with 10 μM monensin and 10 μM nigericin in Living Cell Imaging Solution (Thermo Fisher, USA). The fluorescence intensity was measured at Ex-330/Em-550 and Ex-380/Em-550. The standard curve of the pH value was generated with an Intracellular pH Calibration Buffer Kit (Thermo Fisher, USA).

To measure the relative acidity of lysosomes, macrophages treated with MPs or DMA (Sigma–Aldrich) were incubated with 5 μM LysoSensor^TM^ Green DND-189 (Thermo Fisher, USA) for 30 min under appropriate growth conditions. Then, the loading solution was replaced with a fresh medium, and the stained cells were observed and digitalized utilizing a Nikon A1 confocal microscope. The relative intensity was analyzed with ImageJ 9.0 software.

### Endosomal acidity detection

To detect endosomal acidity, pHrodo^TM^ Red Dextran (Thermo Fisher, USA), which exhibits pH-sensitive fluorescence emission that increases in intensity with increasing acidity and is essentially nonfluorescent in the extracellular environment, was utilized. Following the manufacturer’s guidelines, AMs were cultured with 50 μg/ml pHrodo^TM^ Red Dextran in Live Cell Imaging Solution for 10 min at 37 °C. After washing with prewarmed medium twice, the cells were imaged under a Nikon A1 confocal microscope with an appropriate filter.

### Western blotting

Cells were lysed in M2 lysis buffer and sonicated. The protein concentration was determined with a BCA kit (Applygen Technologies Inc., China). Then, the isolated protein was run on an SDS–PAGE gel and transferred to a nitrocellulose membrane. Nitrocellulose membranes were blocked in 5% bovine serum albumin (BSA) and probed with anti-ACE2 (1:1000, Abcam, Cat. ab108252), anti-lamp2 (1:1000, Abcam, Cat. ab25339), anti-GAPDH (1:2000, CST, Cat. 5174), anti-α-Tubulin (1:2000, Sigma–Aldrich, Cat. T6074) and anti-CH25H (1:200, Santa Cruz Biotechnology, Cat. sc-293256) antibodies overnight. Secondary antibodies conjugated to horseradish peroxidase were added, followed by visualization by enhanced chemiluminescence (Thermo Fisher, MA). The results were confirmed by at least three independent experiments.

### Filipin staining

Cell-generated MPs were stained with 100 μg/mL Filipin III (Sigma, Cat. SAE0087) at 4 °C for 1 h. Then, the MPs were washed with PBS and fixed in paraformaldehyde. The MPs were imaged by ultrahigh-resolution structured illumination microscopy. For cholesterol depletion, MeβCD (5 mg/mL) was added to the cells at 37 °C for 2 h before irradiation.

### 25-HC measurements

To extract oxysterols, 1 mL of EtOH was added to MPs and then sonicated. The extracts were dried under N2 steam. The residue was resuspended in 100 μL of EtOH. Then, the 25-HC in the MPs was analyzed by liquid chromatography-tandem mass spectrometry as described previously [63, 64]. The analysis was carried out on a Waters ACQUITY H-class LC system coupled with a Waters Quattro Micro mass spectrometer, and a Waters Xbridge C18 column (2.1 mm × 100 mm, 3.5 μm) was used. Mobile phase A (H2O/0.1% formic acid) and phase B (MeOH/0.1% formic acid) were applied at a flow rate of 0.3 ml/min. The gradient program was composed of a 15-min linear gradient from 60–97% phase B followed by a 10-min isocratic elution of 97% phase B. The mass spectrometer was operated in the positive ion mode, and 25-HC was quantified using the following MS/MS transitions: *m/z* 385.5 > 367.5 (cone: 15 V, collision energy: 15) and *m/z* 385.5 > 159.2 (cone: 15 V, collision energy: 30).

### Cholesterol assay

According to the manufacturer’s protocol (Promega, Cholesterol/Cholesterol Ester-Glo^TM^ Assay), cells were seeded in a 96-well plate. The next day, the cells were counted and incubated with 50 μL of Cholesterol Lysis Solution for 30 min at 37 °C. Subsequently, 50 μL of Cholesterol Detection Reagent was added. We calculated the amount of free cholesterol by comparison of the luminescence of samples and standards.

### Serum biochemistry

All the steps were performed according to the manufacturer’s protocol. ALT (Cat. c009-2-1), AST (Cat. c010-2-1), and CRE (Cat. c011-2-1) were purchased from Nanjing Jiancheng Bioengineering Institute (China).

### Animal experiments and treatment protocols

Animal studies involving the SARS-CoV-2 strain WH-09 were performed in an animal biosafety level 3 (BASL3) facility using HEPA-filtered isolators, and the procedures were approved by the Institutional Animal Care and Use Committee (IACUC; Protocol Number: ZH20005) of the Institute of Laboratory Animal Science, Peking Union Medical College (BLL20001). Murine studies not involving viral infection were approved by the Animal Care and Use Committee of the Chinese Academy of Medical Sciences. The hACE2 transgenic mouse model used was originally established in Chuan Qin’s laboratory [65]. hACE2 mice were infected with SARS-CoV-2 (1 × 10^5^ TCID_50_) by intranasal administration and then treated with a vehicle control (PBS) or AO-MPs (5 × 10^6^, i.n.) once per day for 5 days (*n* = 5 mice/group). After 5 days of treatment, the mice were euthanized, and lung tissues were collected for real-time PCR analysis and histological and immunohistochemical staining. Upon SARS-CoV-2 infection, the hACE2 mice exhibited overt lung pathological damage on Day 5 but usually survived the infection. For SARS-CoV-2 S pseudovirus (Sino Biological, Cat. PSV001) infection, pseudoviruses (1 × 10^5^ TCID_50_) were preincubated with AO-MPs for 30 min at 37 °C and then used to infect hACE2 mice by intranasal administration. After 30 min, the lung tissues were collected to perform frozen sectioning.

### Quantification and statistical analysis

All experiments were performed at least three times. Results are expressed as the mean ± SD as indicated and were analyzed by a two-tailed Student’s *t-*test or one-way ANOVA followed by Bonferroni’s test. *P* < 0.05 was considered statistically significant. Analyses were conducted using GraphPad 8.0 software.

## Supplementary information

supplementary information

## Data Availability

All data needed to evaluate the conclusions in the paper are presented in the paper or the Supplementary Materials. Materials described in the study are either commercially available or available upon request from the corresponding author.
