# Supplementary material for: SARS-CoV-2 treatment effects induced by ACE2-expressing microparticles are explained by the oxidized cholesterol-increased endosomal pH of alveolar macrophages
Source: Cell Mol Immunol. 2022 Jan 4;19(2):210–21. doi: 10.1038/s41423-021-00813-6 (PMC8724656; doi:10.1038/s41423-021-00813-6)
Supplement: Supplementary file 1 — supplementary information [file 41423_2021_813_MOESM1_ESM.docx]

**Supplementary Information**


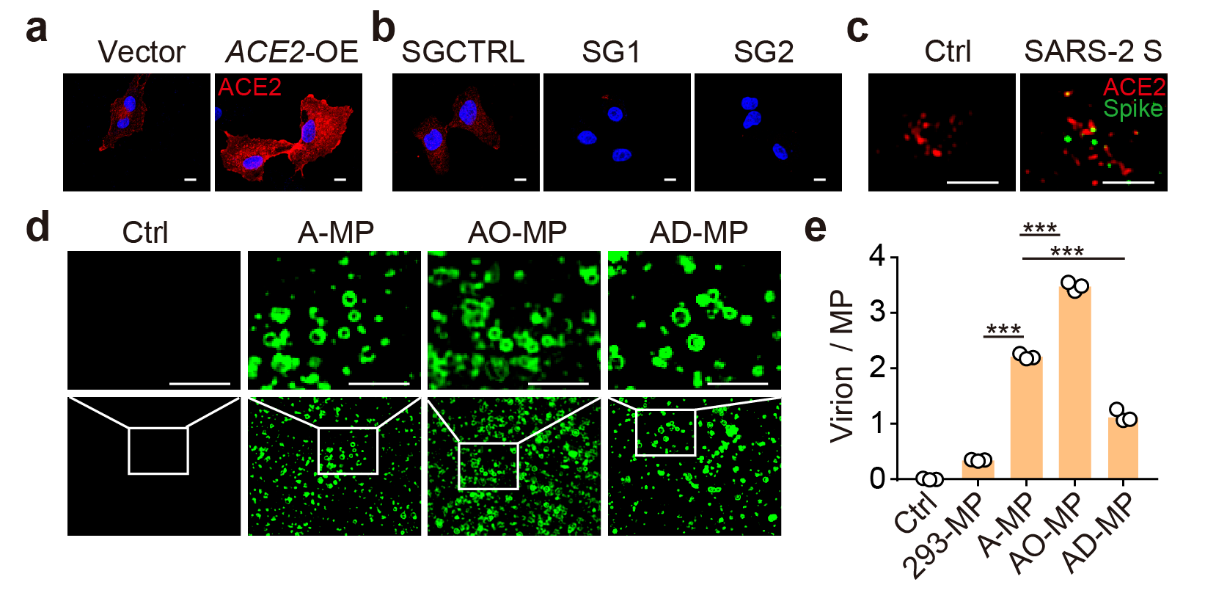


**Supplementary Fig. 1 MPs are able to adsorb SARS-CoV-2. a-b,** The efficiency of *ACE2*-overexpression or *ACE2*-konck out in A549 cells was detected by immunofluorescent staining. Scale bar, 5 μm. **c,** 5×10^5^ A-MPs were incubated with 0.1 μg recombinant SARS-CoV-2 spike protein, and then stained with anti-ACE2 (red) and anti-S protein (green) antibodies. Scale bar, 2 μm. **d,** The A-MPs, AO-MPs, or AD-MPs were labeled with PKH-67 (green), observed by ultra-high-resolution Structured Illumination Microscope. Ctrl, a MP-free control staining. Scale bar, 2 μm. **e,** MPs or PBS (Ctrl) were incubated with SARS-CoV-2 for 30 min at 37 ℃, and then the mixture was filtered through 0.1 μm filter. The virus load (N copy number) on filter membrane was analyzed by real-time PCR. The data represent mean ± SD of three independent experiments. *** *p*<0.001, by two tailed Student's *t*-test (**e**).


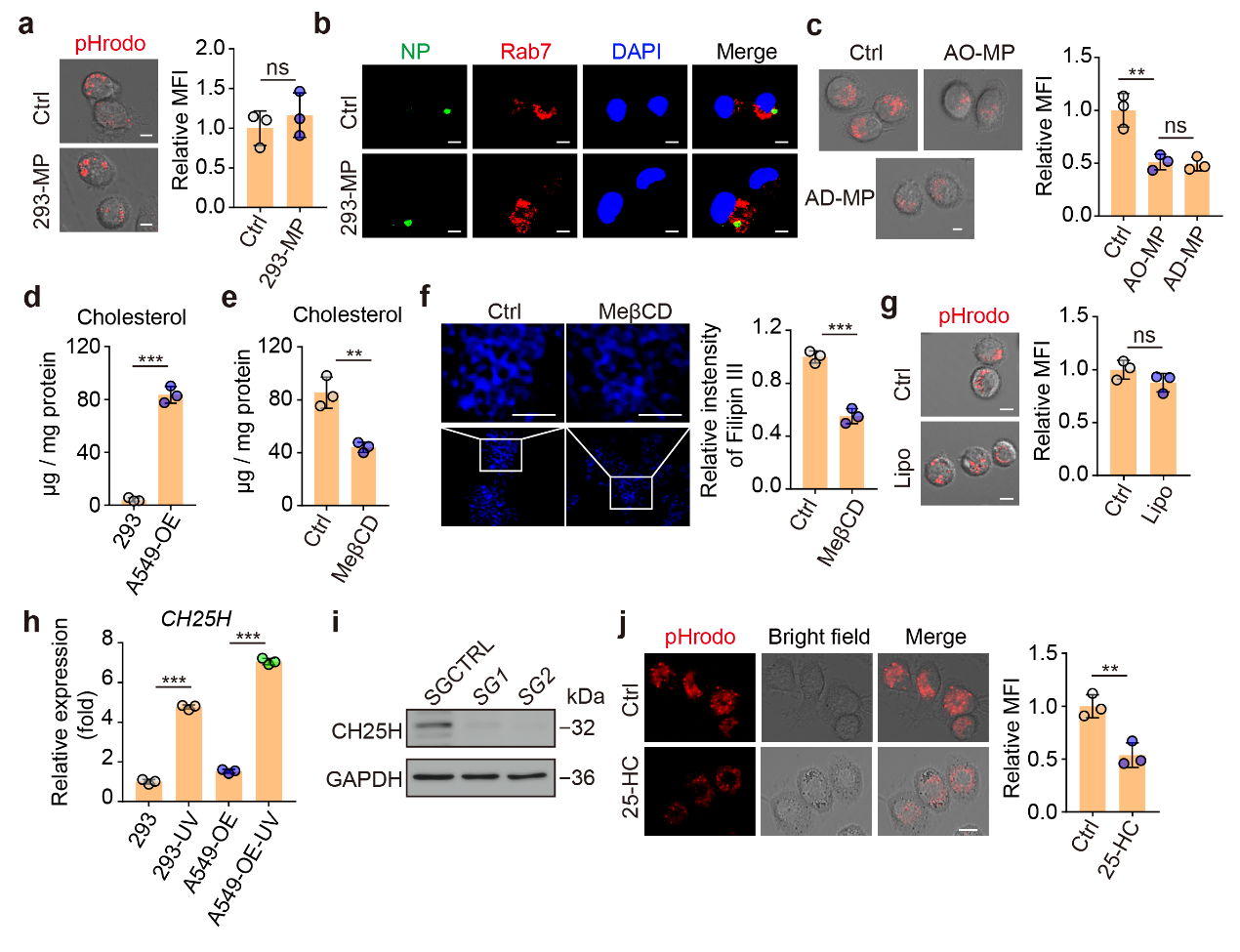


**Supplementary Fig. 2 Neither 293T-derived MPs nor liposomes altered endosomal pH. a, g,** AMs were pretreated with 293-MPs (5 ×10^5^) (**a**) or liposomes (2×10^6^) (**g**) for 30 min, then stained with pHrodo™ Red dextran. Scale bar, 5 μm. **b,** 5 ×10^5^ 293**-**MPs or PBS (Ctrl) were incubated with 5×10^4^ TCID_50_ SARS-CoV-2 for 30 min at 37 ℃, and then infected with AMs for 20 min. Cells were stained with anti-NP and anti-Rab7 antibodies. Scale bar, 5 μm. **c,** AMs were pretreated with AO-MPs (5 ×10^5^) or AD-MPs (5×10^5^) for 30 min, then stained with pHrodo™ Red dextran. Scale bar, 5 μm. **d,** The levels of free cholesterol in 293T and A549-OE cells. **e,** A549-OE cells were pretreated with MeβCD (5 mg/mL) at 37℃ for 2 hr and the levels of free cholesterol were quantified. **f,** The same as (e), except that MPs were stained with Filipin III. Scale bar, 1 μm. **h,** The CH25H expression was detected by qPCR after UV irradiation. **i,** The efficiency of *CH25H*-konck out in A549-OE cells was detected by western blotting. **j,** Primary AMs were treated with 10 μM 25-HC, 12 hours later, the AMs were stained with pHrodo^TM^ Red. Scale bar, 10 μm. The data represent mean ± SD of three independent experiments. ^ns^ no statistical significance, ** *p*<0.01, *** *p*<0.001, by two tailed Student's *t*-test (**a, d-h, j**) or one-way ANOVA (**c**).


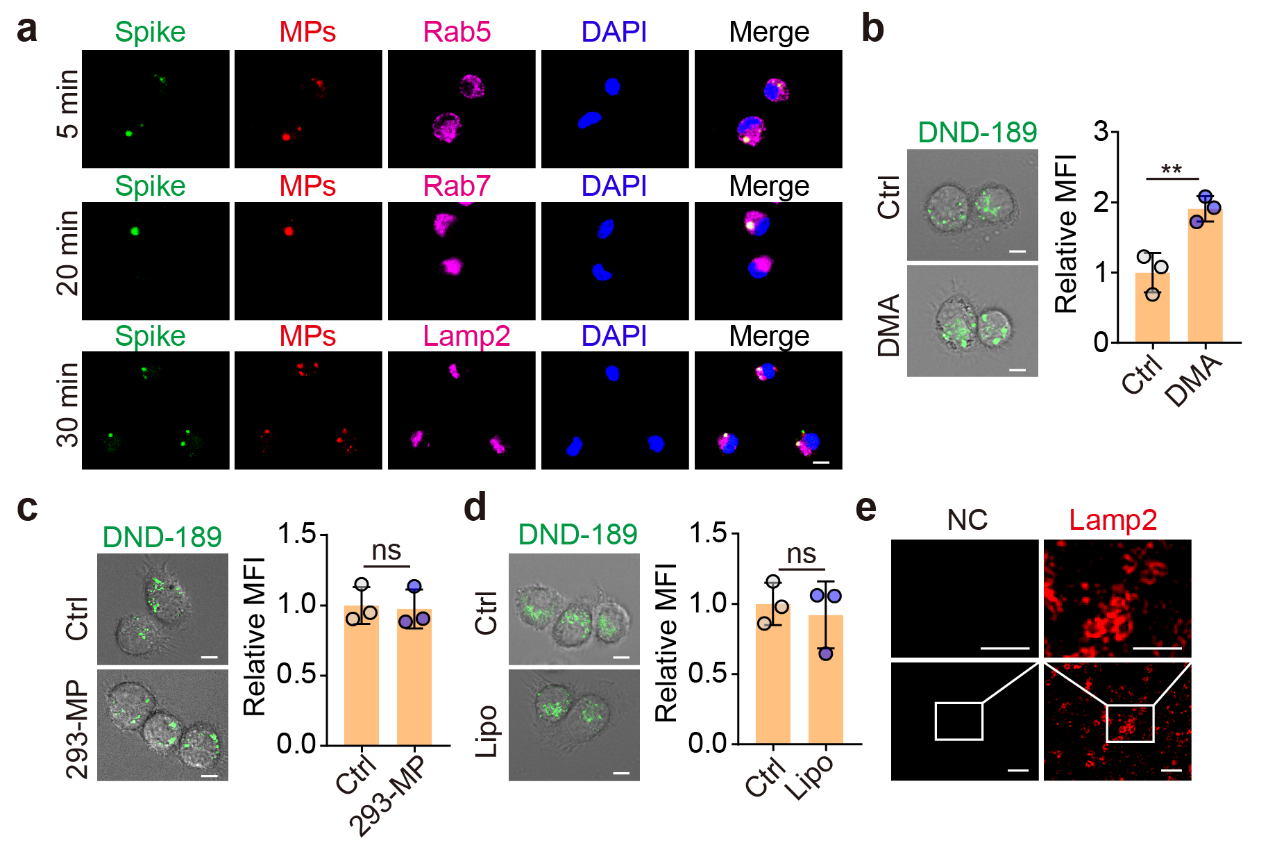
**Supplementary Fig. 3 293T-derived MPs and liposomes did not alter the lysosomal pH.** **a,** 5 ×10^5^ AO-MPs were incubated with 5×10^4^ TCID50 SARS-CoV-2 for 30 min at 37 ℃, and then infected with AMs for 5 min, 20 min or 30 min. Cells were stained with anti-Sipke, anti-Rab5 (a marker for early endosome), anti-Rab7 (a marker for late endosome) and anti-Lamp2 (a marker for lysosome) antibodies. Scale bar, 5 μm. **b-d,** AMs were pretreated with 15 μM DMA (**b**) for 6 hrs, 293-MPs (**c**) and liposomes (**d**) for 30 min, and then stained with LysoSensor™ Green DND-189 for 30 min at 37 ̊C. The cells were observed under confocal microscope. Lipo, liposomes. Scale bar, 5 μm. **e,** Cells were performed to separate lysosomal fraction and cytoplasmic fraction from AO-MPs treated or untreated Raw264.7 cells. The purified lysosomes were detected by immunofluorescence. NC, negative control. Scale bar, 1 μm. The data represent mean ± SD of three independent experiments. ^ns^ no statistical significance, ** *p*<0.01, by two tailed Student's *t*-test (**b-d**).


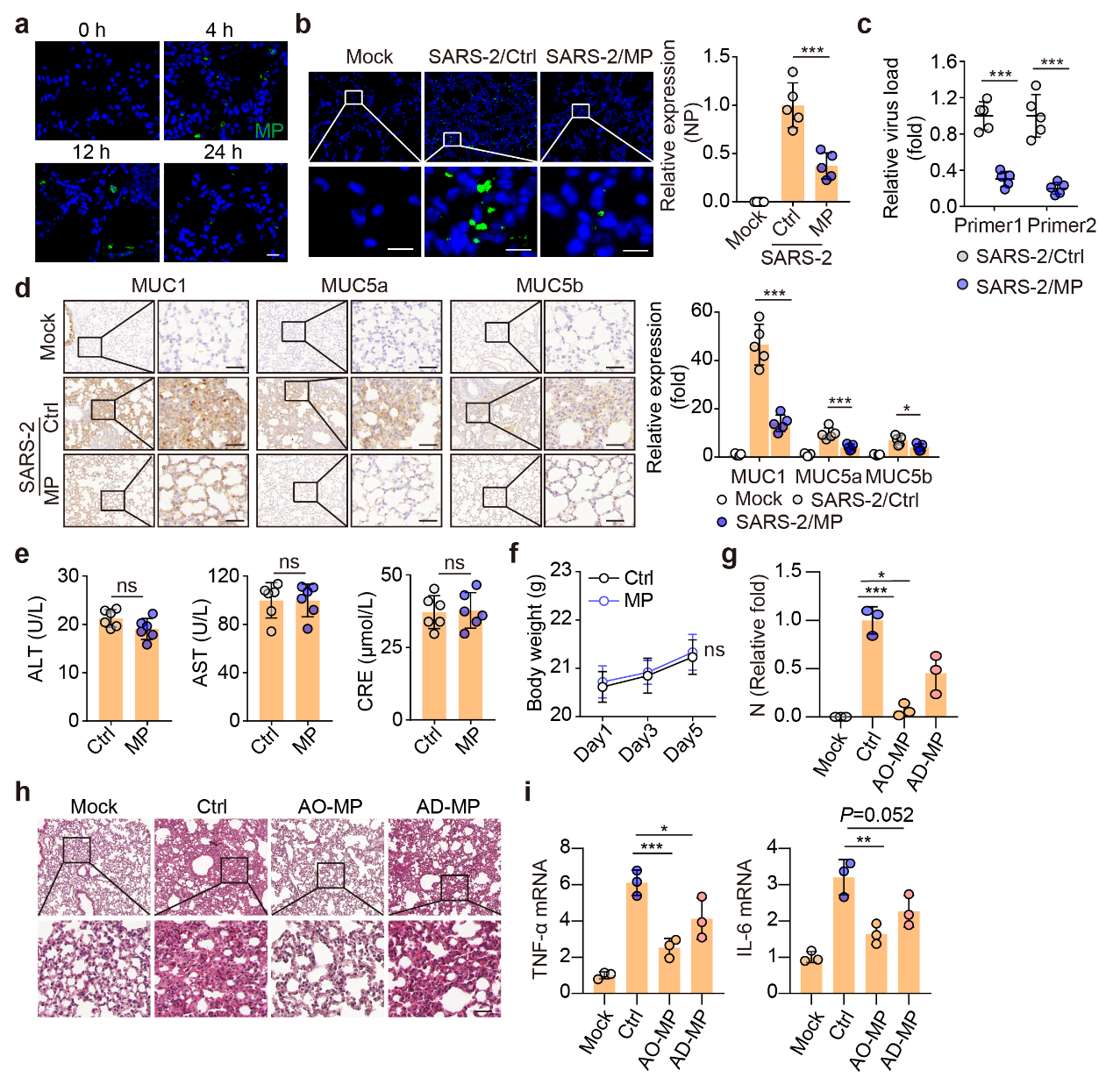


**Supplementary Fig. 4 Treatment effects of AO-MPs against SARS-CoV-2 infection *in vivo*. a,** The ICR mice were intranasally administered PBS (Ctrl) or PKH67-labeled AO-MPs (green) (5×10^6^, 50 μl). The lung tissues were collected to prepare frozen sections at 4 h, 12 h or 24 h. The AO-MPs in lung tissues were observed by confocal microscopy. Scale bar, 20 μm. **b-d,** hACE2-transgenic mice were infected with 1×10^5^ TCID_50_ SARS-CoV-2, and then administered with AO-MPs (i.n., 50 μl, 5×10^6^) once per day for 5 days. The lung tissues were fixed for the immunohistochemical staining of NP (**b**, n = 5) or mucins 1, 5a and 5b (**d**, n=5). The virus load in lung tissues was determined by real-time PCR (**c**). Primer1 targets ORF1ab gene and Primer2 targets N gene. Three lung sections from the left lobe were evaluated for each mouse. The representative images reflected the distributions of damaged lung tissues. **b,** Scale bar, 10 μm. **d,** Scale bar, 50 μm. **e-f,** ICR mice (n=6) were treated with AO-MPs (i.n., 50 μl, 5×10^6^) once per day for 5 days. The levels of ALT (alanine transaminase), AST (aspartate transaminase) or CRE (creatine) in serum were detected (**e**) and the body weight was observed (**f**). **g-i,** hACE2-transgenic mice were infected with 1×10^5^ TCID50 SARS-CoV-2 and at 2 hours post infection, mice were administered with AO-MPs or AD-MPs (i.n., 50 μl, 5×10^6^) once per day for 5 days. The control group (Ctrl) received vehicle (PBS) as placebo. The viral load (**g**) and cytokines (**i**) were determined by real-time PCR. The lung tissues were fixed for H&E staining (**h**). The data represent mean ± SD. ^ns^ no statistical significance, * *p*<0.05, ** *p*<0.01, *** *p*<0.001, by two tailed Student's *t*-test (**b, c, e, f**), one-way ANOVA (**d, g, i**).
